# Oncologic effect of preoperative endoscopic sphincterotomy in patients undergoing pancreaticoduodenectomy for ampulla of vater cancer

**DOI:** 10.1007/s00423-025-03730-8

**Published:** 2025-05-16

**Authors:** Su Min Jeon, Yoo Jin Choi, Hye-Sung Jo, Sae Byeol Choi, Wan-Bae Kim, Hyung-Joon Han, Tae Jin Song, Dong-Sik Kim, Young-Dong Yu

**Affiliations:** 1 Division of HBP Surgery, Department of Surgery, Korea University Anam Hospital, Seoul, Korea; 2 Division of HBP Surgery, Department of Surgery, Korea University Guro Hospital, Seoul, Korea; 3 Division of HBP Surgery, Department of Surgery, Korea University Ansan Hospital, Ansan, Korea; 4 Department of Surgery, Division of HBP Surgery & Liver Transplantation, College of Medicine, Korea University, 73 Goryeodae-ro Seongbuk-gu, Seoul, 02841 Korea

**Keywords:** Endoscopic sphincterotomy, Ampulla of vater, Preoperative biliary drainage, Survival rate

## Abstract

**Purpose:**

Ampulla of Vater (AoV) cancer often presents with bile flow obstruction requiring bile drainage. Endoscopic sphincterotomy (EST) for AoV cancer may result in inflammation and spread of the tumor due to the abundant lymphatic channels and microvascular structures around the AoV, which may impact the postoperative and oncologic outcomes. This study aimed to evaluate the postoperative and oncological effects of EST on AoV cancer.

**Methods:**

Medical records of 131 patients with AoV cancer who underwent pancreaticoduodeenectomy between 2011 and 2020 were reviewed. We compared the perioperative outcomes, overall survival, and disease recurrence in the patients.

**Results:**

Approximately 71 patients underwent EST for preoperative biliary drainage, whereas 60 did not receive any procedure or underwent percutaneous transhepatic biliary drainage. No significant differences were observed in the 5-year overall survival rate (EST 69.9% vs. no EST 75.1%, *P* = 0.804) or the 5-year cumulative recurrence rate (EST 49.1% vs. no EST 56.8%, *P* = 0.855). However, in subgroup analysis using the T stage, EST was associated with reduced 5-year overall survival in the T3 and T4 stages (EST 34.8% vs. no EST 78.0%: *P* = 0.038).

**Conclusion and Discussion:**

Preoperative endoscopic intervention for AoV cancer did not affect oncologic outcomes. However, in the advanced stage, direct manipulation of cancer may result in lower overall survival, requiring careful consideration for preoperative biliary drainage.

## Introduction

Ampulla of Vater (AoV) cancer is a rare form of gastrointestinal malignancy. Owing to its anatomical features, AoV cancer is often associated with obstructive jaundice. In some cases, preoperative biliary drainage (PBD) needs to be performed to decrease hyperbilirubinemia as jaundice is associated with an elevated risk of liver failure, intraoperative blood loss, postoperative bleeding, and infection [1–3]. PBD includes percutaneous biliary drainage (PTBD) and endoscopic retrograde biliary drainage (ERBD). Previous studies demonstrated that PBD is associated with negative short- and long-term outcomes [4–9]. Additionally, PBD may potentially induce iatrogenic cholangitis, pancreatitis, postoperative bleeding, and wound infections.

Some studies demonstrated that ERBD has worse short-term outcomes than those of PTBD. ERBD was related with higher incidence of post procedural complications and higher mortality in patients with cholangiocarcinoma [10, 11]. ERBD often accompanies endoscopic sphincterotomy (EST), which in contrast to PTBD, inevitably irritates the tumor. Abundant lymphatic channels and microvascular structures are distributed throughout AoV [12]. As lymphatic capillaries transport immune cells and can contribute to metastasis by carrying tumor cells, EST may influence cancer spread by directly dissecting AoV cancer cells. Previous studies showed that preoperative ERCP was associated with early distant metastasis and recurrence in patients with AoV cancer and has detrimental effects on long-term survival outcomes [5–6].

To be best of our knowledge, few studies have focused on the long-term, oncologic effects of EST in AoV cancer particularly. The effects of preoperative EST on tumor cell dissemination in patients with AoV cancer have not yet been thoroughly evaluated. However, preoperative EST is routinely performed in patients suspicious of AoV cancer because of obstructive jaundice. This study aimed to evaluate the oncological influence of EST on overall survival and disease recurrence of AoV cancer.

## Methods

### Patients and study design

We retrospectively reviewed the medical records of 131 patients with pathologically confirmed AoV cancer who underwent preoperative biliary drainage followed by pancreaticoduodenectomy at three tertiary centers at the Korea University Medical Center between 2011 and 2020. The median follow-up duration was 31 months. Biliary drainage included ERBD and PTBD. PBD was performed in patients with obstructive jaundice associated with hyperbilirubinemia. Regarding the type of PBD, our institution generally prefers endoscopic retrograde biliary drainage (ERBD), as it is an internal drainage method and does not require patients to carry an external catheter bag, unlike percutaneous transhepatic biliary drainage (PTBD).

While EST is commonly performed as part of endoscopic retrograde cholangiopancreatography (ERCP), it is not a required procedure for ERCP. Since this study focused on the role of EST, patients were divided into two groups based on whether they had undergone EST for biliary drainage: (1) patients who underwent EST for biliary drainage during ERCP procedure, and (2) patients who did not undergo EST or who underwent PTBD before surgery. Information on sphincterotomy was provided in the medical report. Patients who underwent endoscopic retrograde cholangiopancreatography (ERCP) and whose medical records included EST were included in the EST subgroup.

Patient demographics, perioperative outcomes, and tumor histopathology were analyzed, and the overall survival and recurrence rates were compared. Perioperative outcomes included operation time, estimated blood loss, postoperative complications, Clavien–Dindo classification, postoperative pancreatic fistula (POPF), and postoperative day. The primary tumor (T) and regional lymph node (N) categories were based on the 8th edition of the American Joint Committee on Cancer staging system [13]. This study was approved by the Institutional Review Board of the Korea University Medical Center (No.2024AN0232).

### Statistical analysis

Statistical analyses were performed using SPSS version 26.0 (SPSS Corp., Armonk, NY, USA). Between-group comparisons were performed using independent t-tests. Overall and disease-free survival rates were calculated using the Kaplan–Meier method, and survival curves were compared using the log-rank test. Statistical significance was set at *P* < 0.05. Multivariate analysis was conducted using Cox proportional hazard regression. Two-sided p-values of < 0.05 were considered statistically significant.

## Results

### Demographics, operative, and pathologic features

The mean age of the 131 patients was 49.5 years, with 83 (63.4%) being men. In the study, PBD was performed on 91 patients (69.5%), meanwhile, 40 (30.5%) underwent upfront surgery without drainage. Among those who underwent PBD, only 4 patients underwent PTBD, which was considered to have a less significant impact on the outcomes. Four patients underwent PTBD, two had the procedure performed at other hospitals before presenting to ours, and two underwent PTBD due to difficulties with cannulation caused by duodenal stenosis and a history of gastrectomy. We have revised the Methods section accordingly to clarify these points. Of 87 patients who underwent ERCP, 71 underwent EST before surgery. We included patients who did not undergo PBD (*N* = 40) and EST during ERCP (*N* = 16), along with those who did undergo PTBD (*N* = 4) in the “no-EST” group (*N* = 60). The mean age of EST group was 67.3 year, higher than that of no-EST group, 63.8 year (*P* = 0.041). With the exception of age, no statistically significant differences in other demographic characteristics between the two groups were observed. Notably, the total bilirubin level at diagnosis was not significantly different between the two groups (5.7 mg/dL in the EST group and 4.2 mg/dL in the no-EST group; *P* = 0.192). (Table 1) The reason the total bilirubin level at diagnosis did not significantly differ between the EST and no-EST groups is that patients were grouped based on whether EST was performed, not on whether they underwent PBD. Not all patients who underwent ERCP received EST. In addition, four patients underwent PTBD for biliary drainage.

**Table 1:** Demographics features, operative outcomes and pathologic outcomes

	Total (*N* = 131)	EST (*N* = 71)	No EST (*N* = 60)	*P*-value
Age (yr)	65.7 ± 10.0	67.3 ± 8.8	63.8 ± 11.0	0.041
Sex, male (%)	83 (63.4%)	47 (66.2%)	36 (60.0%)	0.463
ASA I, II (%)	121 (92.3%)	65 (91.6%)	56 (93.4%)	0.542
Total bilirubin, at diagnosis (mg/dL)	5.1 ± 6.7	5.7 ± 7.1	4.2 ± 6.1	0.192
Adjuvant therapy				
Chemotherapy	49 (37.4%)	23 (32.4%)	26 (43.3%)	0.197
Radiotherapy	14 (10.7%)	8 (11.3%)	6 (10.0%)	0.815
Operation time (min)	368.2 ± 127.2	379.4 ± 105.5	369.9 ± 140.7	0.671
EBL, (ml)	589.3 ± 493.5	687.2 ± 567.4	482.3 ± 367.5	0.036
Postoperative complication	52 (39.7%)	29 (40.8%)	23 (38.8%)	0.770
Clavien-Dindo classification ≥ IIIa	43 (31.0%)	21 (29.6%)	20 (33.4%)	0.593
POPF (grade B)	28 (21.4%)	17 (23.9%)	11 (18.3%)	0.435
Postoperative day	27.1 ± 19.0	29.5 ± 21.3	25.3 ± 15.9	0.209
Mass size	2.2 ± 1.3	2.1 ± 1.3	2.4 ± 1.2	0.156
T stage				0.465
T1	48 (35.6%)	28 (39.4%)	17 (28.3%)	
T2	40 (29.6%)	21 (29.6%)	18 (30.0%)	
T3	40 (29.6%)	18 (25.4%)	22 (36.7%)	
T4	7 (5.2%)	4 (5.6%)	3 (5.0%)	
N stage				0.258
N0	88 (67.2%)	51 (71.8%)	37 (61.7%)	
N1	39 (29.8%)	17 (23.9%)	21 (36.7%)	
N2	4 (3.2%)	3 (4.2%)	1 (1.7%)	
Microscopic lymphatic inv.	49 (37.4%)	23 (32.4%)	26 (43.3%)	0.197
Microscopic vascular inv.	17 (13.0%)	10 (14.1%)	7 (11.7%)	0.674
Microscopic perineural inv.	26 (19.8%)	14 (19.7%)	12 (20.0%)	0.968

ASA, American society of anesthesiologist

EBL, estimated blood loss

POPF, post operative pancreatic fistula

The mean operation time was 379.4.1 min for the EST group and 369.9 min for the no-EST group (*P* = 0.671). No significant differences were observed in postoperative complications, Clavien–Dindo classification, POPF, or postoperative day between the two groups. Additionally, there was no significant difference in the administration of postoperative chemotherapy between the two groups. (Table 1). One patient died after pancreaticoduodenectomy in hospital because of pulmonary edema, which led to ICU and ventilator care. However, the mean estimated blood loss was higher in the EST group (687.2 mL) than in the no-EST group (482.3 mL) (*P* = 0.036).

No significant differences were noted in the two groups regarding pathologic outcomes, including lymphatic (EST 32.4% vs. no EST 43.3%; *P* = 0.197), vascular (EST 14.1% vs. no-EST 11.7%; *P* = 0.674), and perineural (19.7% vs. 20.0%; *P* = 0.968) invasion. (Table 1)

### Comparison of oncologic outcomes

**Fig. 1:**
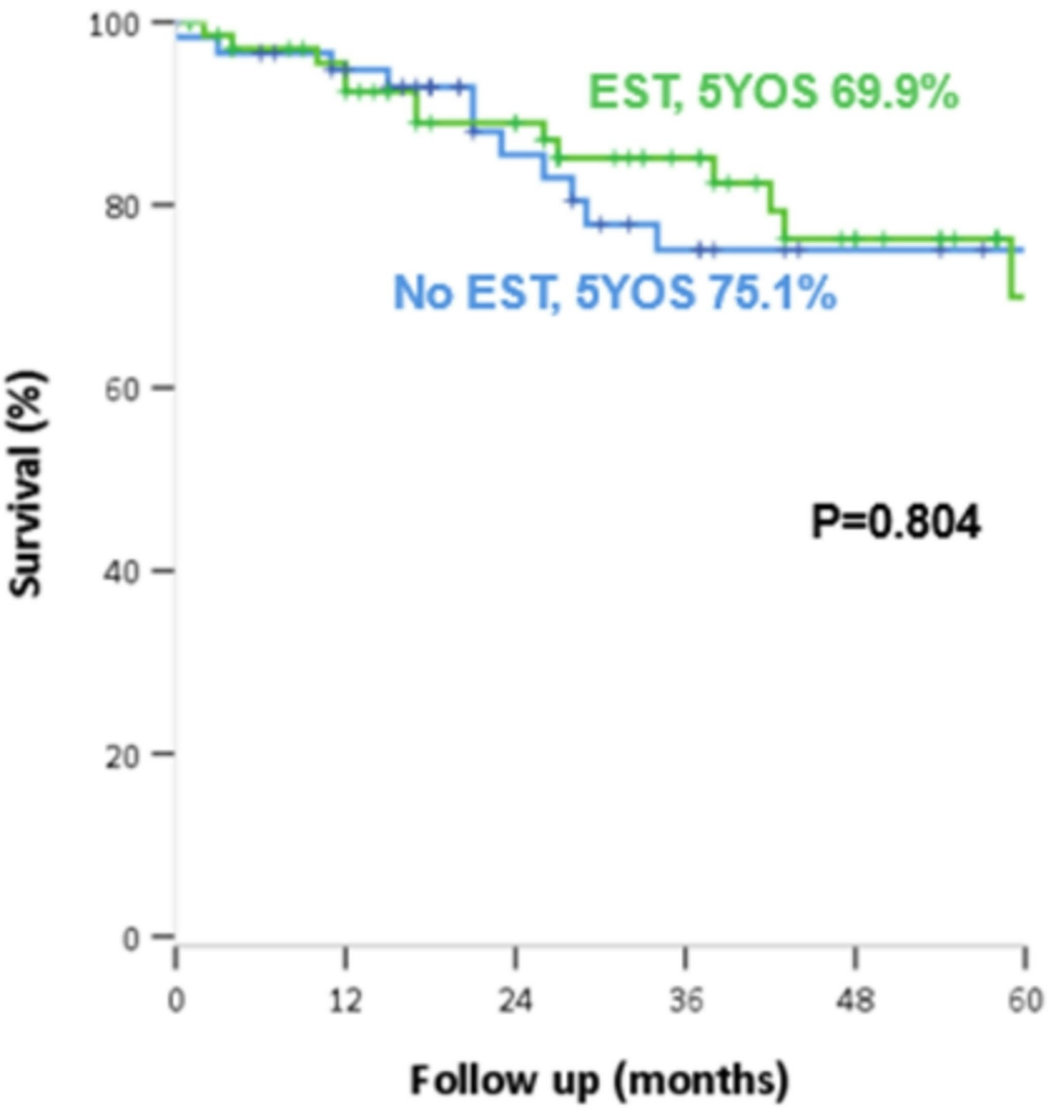
Overall survival according to whether or not preoperative endoscopic sphincterotomy (EST) was performed

**Fig. 2:**
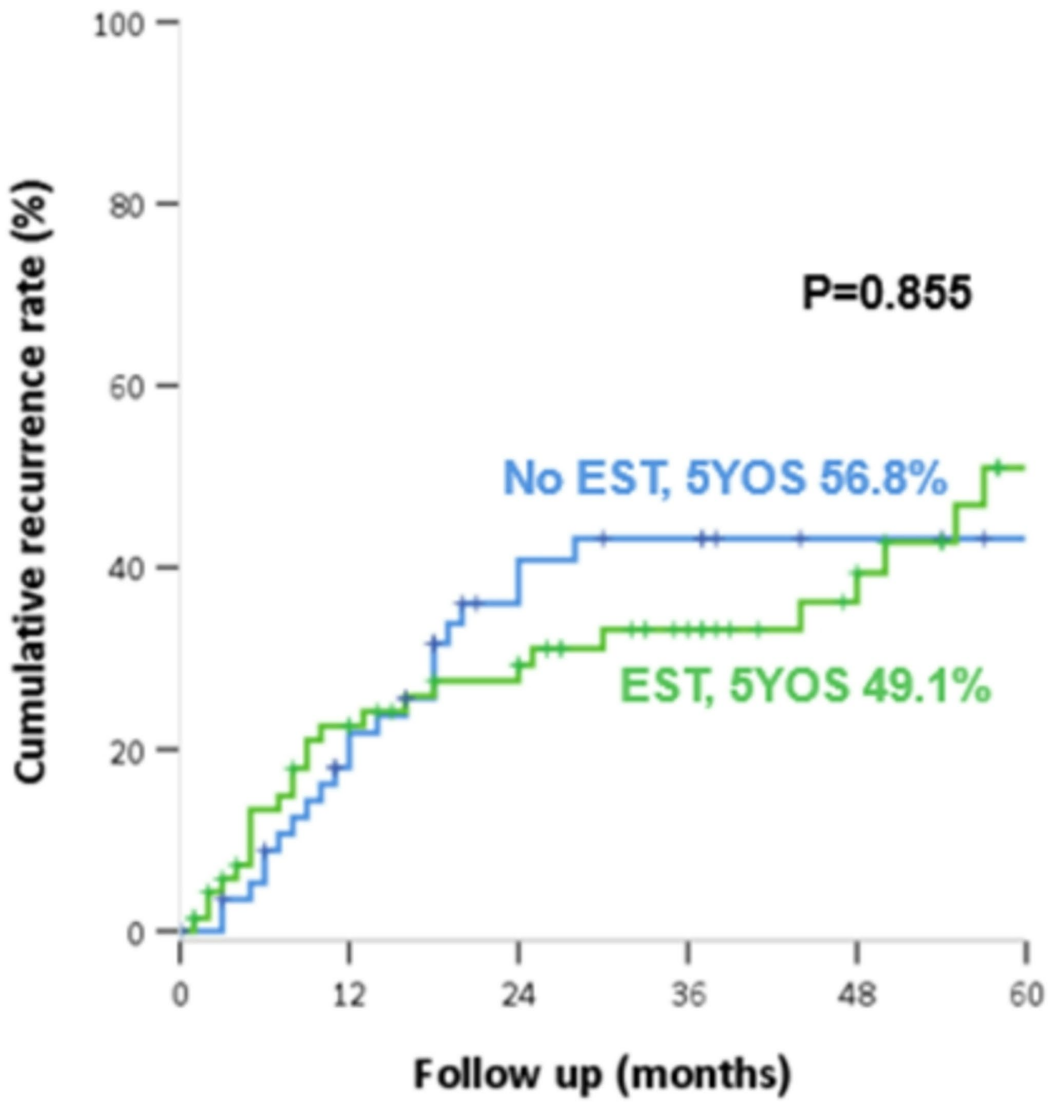
Recurrence according to whether or not preoperative endoscopic sphincterotomy (EST) was performed

The median follow-up duration was 31 months. No significant difference was observed between the two groups in the 5-year overall survival rates (EST 69.9% vs. no EST 75.1%, *P* = 0.804) (Fig. 1) and in the 5-year cumulative recurrence rate (EST 49.1% vs. no EST 56.8%, *P* = 0.855) (Fig. 2), respectively in EST vs. no-EST groups.

Moreover, in subgroup analysis using the T stage, overall survival and recurrence free survival were compared in patients with early-stage (T1 and T2) and with advanced-stage (T3 and T4). The early-stage cancer, T1, and T2 had no significant difference in overall survival and recurrence-free survival; however, in the T3 and T4 stages, the no-EST group had significantly longer overall survival than that observed in the EST group (EST 34.8% vs. no EST 78.0%, *P* = 0.038) (Table 2).

**Table 2:** Oncologic outcomes according to T stage

	T1, T2			T3, T4			
	EST = 49	No EST = 35	*P*-value	EST = 22	No EST = 25	*P*-value	
5Y-OS	82.4%	72.7%	0.245	34.8%	78.0%	0.038	
5Y-RSF	53.1%	66.7%	0.965	39.3%	44.5%	0.815	

OS, overall survival; RSF, recurrence free survival; EST, endoscopic sphincterotomy

In terms of recurrence patterns, no significant difference was observed in the cumulative recurrence rate and recurrence location (Table 3).

**Table 3:** Recurrence outcomes

	Total (*N* = 131)	EST (*N* = 71)	No EST (*N* = 60)	*P*-value
Recur	51 (37.8%)	26 (36.6%)	24 (40.0%)	0.692
Recur location				
Liver	19 (52.8%)	9 (60.0%)	9 (45.0%)	0.853
Local	8 (22.2%)	3 (20.0%)	5 (25.0%)	
Lymph node	5 (13.9%)	2 (13.3%)	3 (15.0%)	

Multivariate Cox proportional hazards analysis was performed to identify potential prognostic factors associated with survival in patients with AoV cancer (Table 4). Among the variables analyzed, T4 stage (hazard ratio, 6.084, *P* = 0.026), microscopic vascular invasion (hazard ratio, 6.030, *P* = 0.009) and recurrence (hazard ratio, 7.589, *P* = 0.000) emerged as significant predictors of survival.

**Table 4:** The prognostic factors for ampulla of vater cancer in the Cox-proportional hazard model

Variable		HR	95% CI	*P*-value
Age	≥ 65 year	1.414	0.555–3.603	0.467
Chemotherapy		1.025	0.375-2.800	0.961
Postoperative complication		1.865	0.728–4.783	0.194
T stage	T1	1	Ref	
	T2	0.859	0.244–3.023	0.813
	T3	1.509	0.510–4.463	0.457
	T4	6.084	1.240-29.841	0.026
Lymph node metastasis		2.288	0.816–6.414	0.116
Microscopic lymphatic inv.		0.440	0.140–1.382	0.160
Microscopic vascular inv.		6.030	1.569–23.174	0.009
Microscopic perineural inv.		0.769	0.200-2.952	0.702
Recurrence		7.589	2.593–22.212	0.000
EST		0.688	0.273–1.735	0.688

EST, endoscopic sphincterotomy

## Discussion

AoV cancer, a rare hepatobiliary disease, is often diagnosed with jaundice due to obstruction of the biliary system. Therefore, in many cases, biliary drainage is routinely performed before surgical resection. The prognosis of AoV cancer is more favorable, with a survival rate of 51.1–61% [14–16] compared to the prognosis of other periampullary cancers when R0 resection is performed. However, if lymph node metastasis occurs, which is a significant prognostic factor, the overall survival rate decreases by 48.5% [17]. Based on these facts, this study was primarily designed to evaluate the oncological effect of EST in AoV cancer. Additionally, EST directly impacts the cancer in the AoV, possibly causing preoperative inflammation and lymph node micrometastasis.

Many previous studies have considered the impact of PBD on postoperative short-term outcomes, including complications, postoperative mortality, and length of hospital stay. However, the results of these studies were inconsistent. Some meta-analyses have demonstrated that PBD yields comparable results in terms of postoperative mortality and complications in patients with malignant obstructive jaundice [18, 19]. Conversely, a systematic review by Moole et al. [20] indicated that PBD was associated with fewer major adverse effects compared to upfront surgery, while hospital stay and mortality rates remained comparable between the groups. In contrast, Celotti et al. [4] reported that PBD for hilar cholangiocarcinoma was associated with high postoperative morbidity and an increased rate of wound infection. These conflicting results have led to an ongoing debate regarding the efficacy and safety of PBD in periampullary diseases.

Although these studies mainly discussed cholangiocarcinoma, few have discussed the postoperative oncologic outcomes in patients with AoV cancer who underwent PBD. Byun et al. [9] reported that PBD in patients with AoV cancer adversely affects oncological outcomes, resulting in a reduced 5-year disease-free survival rate and increased lymph node metastasis. By including both ERBD and PTBD in the PBD group and comparing them to upfront surgery, their study demonstrated that any form of PBD could negatively influence the long-term oncologic outcomes in AoV cancer. They also compared ERBD and PTBD in a subgroup analysis, revealing that endoscopic biliary drainage (EBD) had a high rate of complications but no significant difference in disease-free survival. Ahn et al. [6] demonstrated that preoperative ERCP was associated with early distant metastasis in early-stage cancer and was an independent risk factor for recurrence in patients with AoV cancer. Another study by Barauskas et al. [5] reported that routine preoperative endoscopic biliary drainage should be avoided in patients with resectable ampullary carcinoma as preoperative EBD has detrimental effects on long-term survival outcomes. However, these studies have not revealed why EBD negatively affects survival.

Therefore, we focused on the effect of EST in AoV cancer, as the disruption of the tumor prior to surgery could influence its dissemination. This study was confined to patients with pure AoV cancer and explicitly focused on the effect of EST, which involves direct manipulation of the AoV. While many previous studies focused on the presence or absence of preoperative biliary drainage and the differences between ERBD and PTBD, our study classified groups based on whether EST was performed.

Few studies have reported long-term adverse effects of EST. First, interruption of the sphincter of Oddi has been reported to cause bacterial overgrowth in the common bile duct, resulting in cholangitis [21, 22]. Hakamada et al. reported that chronic cholangitis may result in the late development of bile duct cancer after sphincterotomy [23]. In this study, the mean estimated blood loss during the operation was higher in the EST group than in the no-EST group, which might have been attributed to cholangitis and pancreatitis following EST. This inflammatory response could have led to increased vascularity and tissue friability, leading to bleeding during dissection around the bile duct and pancreas. Additionally, based on clinical observations, patients who underwent endoscopic procedures often showed more inflammation. However, EST did not influence postoperative complications. No statistically significant differences were noted in complication rates, incidence of POPF over grade B, or length of postoperative hospital stay between the EST and no-EST groups. These findings suggest that EST does not contribute significantly to severe inflammation.

Second, the microanatomical structure of the AoV, which is reported to have abundant lymphatic connections and blood vessel distribution, may also provide evidence of tumor cell spread through the lymphatic channels and bloodstream after sphincterotomy [12]. EST may facilitate tumor cell dissemination by stimulating widespread lymphatic capillaries, potentially leading to an increased recurrence risk. According to the previous studies, tumor seeding along the catheter tract is unusual but possible, through cellular disruption and dissemination of tumor cells when the tumor is directly manipulated using a guidewire or catheter [24]. Takahashi et al. reported 23 cases (5.2%) of PTBD catheter tract recurrence in patients with resected cholangiocarcinoma and poor prognosis of these recurred patients [25]. However, contrary to our expectations and previous findings, this study did not confirm the hypothesis that EST influences tumor spread in AoV cancer. The data revealed no statistically significant differences in N stage, microscopic lymphatic invasion, or vascular invasion (*P* = 0.305, 0.202, and 0.674, respectively). Moreover, the 5-year overall survival and 5-year recurrence-free survival rates were comparable between the two groups. This lack of difference may be attributed to several factors. First, EST may not have induced inflammation to a degree that would negatively impact oncologic outcomes. Second, the interval between EST and surgical resection was less than 14 days, which may not have provided sufficient time for any potential cancer spread to occur. The interval was not specified in advance and to expedite cancer surgery, operations were performed as soon as the total bilirubin levels decreased below a certain threshold. A study by Shin et al. showed that early surgery performed in the first 2 weeks did not increase postoperative complications while delayed surgery beyond 4 weeks showed worse prognosis [26]. 

Subgroup analysis using the T stage revealed no significant difference in 5-year overall survival between the two groups in the T1 and T2 stages. However, EST was associated with reduced 5-year overall survival in the T3 and T4 stages. These results suggest that EST might adversely affect oncological outcomes in patients with higher-stage AoV cancer because when cancer progresses, tumor manipulation may more greatly promote inflammation and tumor proliferation, provided blood vessels and lymphatics are more developed around the tumor. However, a study by Byun et al. demonstrated that the prognosis of the PBD group was worse than that of the upfront surgery group in patients with T1 and T2 AoV cancer [9]. 

Our findings suggest that preoperative EST does not significantly impact postoperative complications or long-term oncologic outcomes, particularly in patients with early-stage AoV cancer. However, in this subgroup, EST may contribute to increased intraoperative blood loss, likely due to inflammatory changes and increased vascularity around the bile duct and pancreas following endoscopic manipulation. Given these potential effects, careful selection of patients for preoperative biliary drainage is essential to minimize unnecessary procedural risks. In contrast, in patients with advanced-stage disease, direct manipulation of the tumor during EST may negatively influence overall survival, highlighting the need for a more cautious approach in this population. As our results differ from some previous studies, further large-scale investigations are warranted to clarify the oncologic impact of EST, particularly in relation to tumor stage and surgical outcomes.

In the present study, multivariate Cox proportional hazards analysis revealed that T4 stage, microscopic vascular invasion, and recurrence were significant independent predictors of poor overall survival in patients with AoV cancer. These findings are in line with previous reports that have identified the advanced T stage as a critical prognostic factor. For instance, two prior studies demonstrated that a higher T stage is independently associated with decreased survival in patients with AoV cancer. [27–28] Additionally, the prognostic significance of vascular invasion observed in our analysis is supported by earlier research indicating that venous invasion is an independent predictor of worse outcomes in AoV cancer [29]. 

This study had several limitations. First, as this was a retrospective study, a risk of hidden confounders exists that may not have been considered. In addition, as bile duct cancer is a rare malignancy with a low expected incidence, even in a large cohort, it reduces the precision of long-term cancer risk analyses and limits subgroup analyses. However, this is a flaw that the present study shares with all previously published studies concerning EST and cancer. To date, the present study has included a considerable number of patients and long-term oncologic outcomes. Moreover, ERCP or ERBD without EST can irritate the surrounding tissues of the AoV. Considering that the no-EST group also included these cases, this might have affected the results of the two groups.

## Conclusion

Preoperative EST for AoV cancer did not affect long-term oncologic outcomes in terms of overall survival or disease recurrence rates. However, in the advanced stage, direct manipulation of cancer may result in lower overall survival, requiring careful consideration for preoperative biliary drainage. As our results were inconsistent with those of previous studies, further evaluation with extensive data is necessary to assess the direct manipulation of the AoV.

## Data Availability

No datasets were generated or analysed during the current study.
